# Evidence of haptoglobin in the porcine female genital tract during oestrous cycle and its effect on in vitro embryo production

**DOI:** 10.1038/s41598-021-90810-6

**Published:** 2021-06-08

**Authors:** Francisco A. García-Vázquez, Carla Moros-Nicolás, Rebeca López-Úbeda, Ernesto Rodríguez-Tobón, Ascensión Guillén-Martínez, Jason W. Ross, Chiara Luongo, Carmen Matás, Iván Hernández-Caravaca, Manuel Avilés, Mª José Izquierdo-Rico

**Affiliations:** 1 Departamento de Fisiología, Facultad de Veterinaria, Universidad de Murcia, 30100 Murcia, Spain; 2 Departamento de Biología Celular e Histología, Facultad de Medicina, Universidad de Murcia, 30100 Murcia, Spain; 3 Department of Animal Science, Iowa State University, Ames, IA USA; 4 Instituto Murciano de Investigación Biosanitaria (IMIB), Murcia, Spain; 5 CEIR Campus Mare Nostrum (CMN), Murcia, Spain

**Keywords:** Animal biotechnology, Cell biology

## Abstract

Recent evidence supports involvement of the acute phase protein haptoglobin in numerous events during mammalian reproduction. The present study represents an in-depth investigation of haptoglobin expression and secretion in the porcine oviduct and uterus, and assesses its effect on porcine in vitro embryo production. A systematic study was made of sows in different oestrous stages: late follicular, early luteal and late luteal stages. Relative haptoglobin mRNA abundance was quantified by RT-qPCR. In addition, expression of the protein was analysed by immunohistochemistry and the results were complemented by Western-blot and proteomic analyses of the oviductal and uterine fluids. In vitro porcine fertilization and embryo culture were carried out in the presence of haptoglobin. The results indicate that haptoglobin mRNA expression in the porcine oviduct and uterus is most abundant during the late luteal stage of the oestrous cycle. By means of Western blot and proteomic analyses haptoglobin presence was demonstrated in the oviduct epithelium and in the oviductal and uterine fluids in different stages of the oestrous cycle. The addition of haptoglobin during gamete co-incubation had no effect on sperm penetration, monospermy or efficiency rates; however, compared with the control group, blastocyst development was significantly improved when haptoglobin was present (haptoglobin: 64.50% *vs.* control: 37.83%; p < 0.05). In conclusion, the presence of haptoglobin in the oviduct and uterus of sows at different stages of the oestrous cycle suggests that it plays an important role in the reproduction process. The addition of haptoglobin during in vitro embryo production improved the blastocyst rates.

## Introduction

The female reproductive tract, including its secretions, plays a crucial role in several reproductive events, such as the final maturation and transport of gametes, fertilization, early embryo development and implantation^1–6^. Oviductal and uterine fluids are formed by plasma-derived constituents and proteins and other molecules synthetized by the epithelial cells and the uterine glands^6–12^. Previous transcriptomic analyses have provided information about the genes expressed by the porcine oviduct^13–15^ and uterus^16–18^. In pigs, numerous proteins have been identified in the oviductal epithelial cells^19,20^, in oviductal secretions^7,14,21^ and the endometrium^22–24^. Most of these proteins are known to influence the maturation, viability and function of gametes, embryo development and endometrial receptivity, and there is evidence to suggest these proteins prepare the reproductive tract environment for embryo arrival^14,21,22,24^. Increasing our knowledge of the function of these proteins might be useful for improving assisted reproductive techniques (ARTs). In pigs, the developmental competence of in vitro-produced (IVP) embryos is low compared with that of in vivo-produced embryos^25,26^. Indeed, in vivo-produced embryos are better in terms of cryotolerance, morphology, pregnancy rates and epigenetic profile than their in vitro-produced counterparts^25–28^. Moreover, a high incidence of polyspermy occurs in porcine in vitro fertilization (IVF). Different factors could be responsible for this low efficacy, such as an insufficient cytoplasmatic ability for oocyte development, an excessive number of acrosome-reacted spermatozoa at the time of fertilization, or unsuitable culture conditions (reviewed in^29,30^). The differences between the in vivo factors provided by the oviduct and uterus and those of the culture medium affect the success of ARTs^26^. Hence the importance of in-depth analyses of oviductal fluid and uterine fluid proteins.

Haptoglobin, first identified in 1939^31^, is synthesized in the liver and is known to bind free hemoglobin released from erythrocytes with a high degree of affinity, thereby inhibiting its oxidative activity. Moreover, haptoglobin has traditionally been considered as an acute phase protein^32–35^ that acts within the physiological context of an inflammatory response, since it is present during infection, inflammation, traumatic damage and malignant proliferation. Furthermore, haptoglobin has been proposed as a suitable welfare biomarker for monitoring the post vaccination inflammatory response in piglets^36^. On the other hand, additional information is provided by knock-out mice for this gene. These animals are viable and fertile, although there is a small but significant adverse effect on the postnatal viability of mice, suggesting that the hemoglobin-haptoglobin complex plays an important role in the amelioration of tissue damage caused by lipid peroxidation^37^.

However, haptoglobin is now recognized as a normal constituent of healthy mammalian reproductive tissues and fluids, and the reproductive tissues of several species have been shown to express haptoglobin mRNA in the absence of inflammation. For instance, it is expressed in the ovaries of mice^38^, rats^39^ and cows^40^, the oviduct of cows^40,41^ and rabbits^42^ and the endometrium of mice^38^, rabbits^42–44^, humans^45^ and pigs^24^, and in the testes of rats^39^. Moreover, it has been detected in the ovarian follicular fluid from humans^46,47^, water buffalos^48^, and cows^40^, in the oviductal fluid of rabbits^42^, cows^40,49^ and pigs^21^, and in the uterine fluid of rabbit^42^, rat^50^, bonnet monkey^50^ and human^50–53^, among others. Haptoglobin was found to be more abundant in rabbit and human uterine fluid than in serum^42,50^. The presence of haptoglobin in the reproductive organs appears to be specific to certain cell types and/or stages of the reproductive cycle, suggesting that haptoglobin contributes to mammalian reproductive events. However, the physiological function of haptoglobin within the reproductive tissues remains unclear^41^.

Its presence in the oviduct and uterus might indicate that haptoglobin plays a relevant role in reproduction, maybe modulating the interaction of gametes or affecting early embryonic development. In light of the above, the aim of this study was to analyze the following in porcine: (1) the relative abundance of mRNA codifying for haptoglobin protein in the oviduct and uterus (non-pregnant and pregnant sows), (2) the presence of the protein in oviductal fluid and uterine fluid in different phases of the oestrous cycle, (3) the localization of haptoglobin protein in the oviduct during the oestrous cycle, (4) the haptoglobin function by means of functional analyses during in vitro embryo production.

## Results

### mRNA haptoglobin levels are higher during late luteal phase

**Figure 1:**
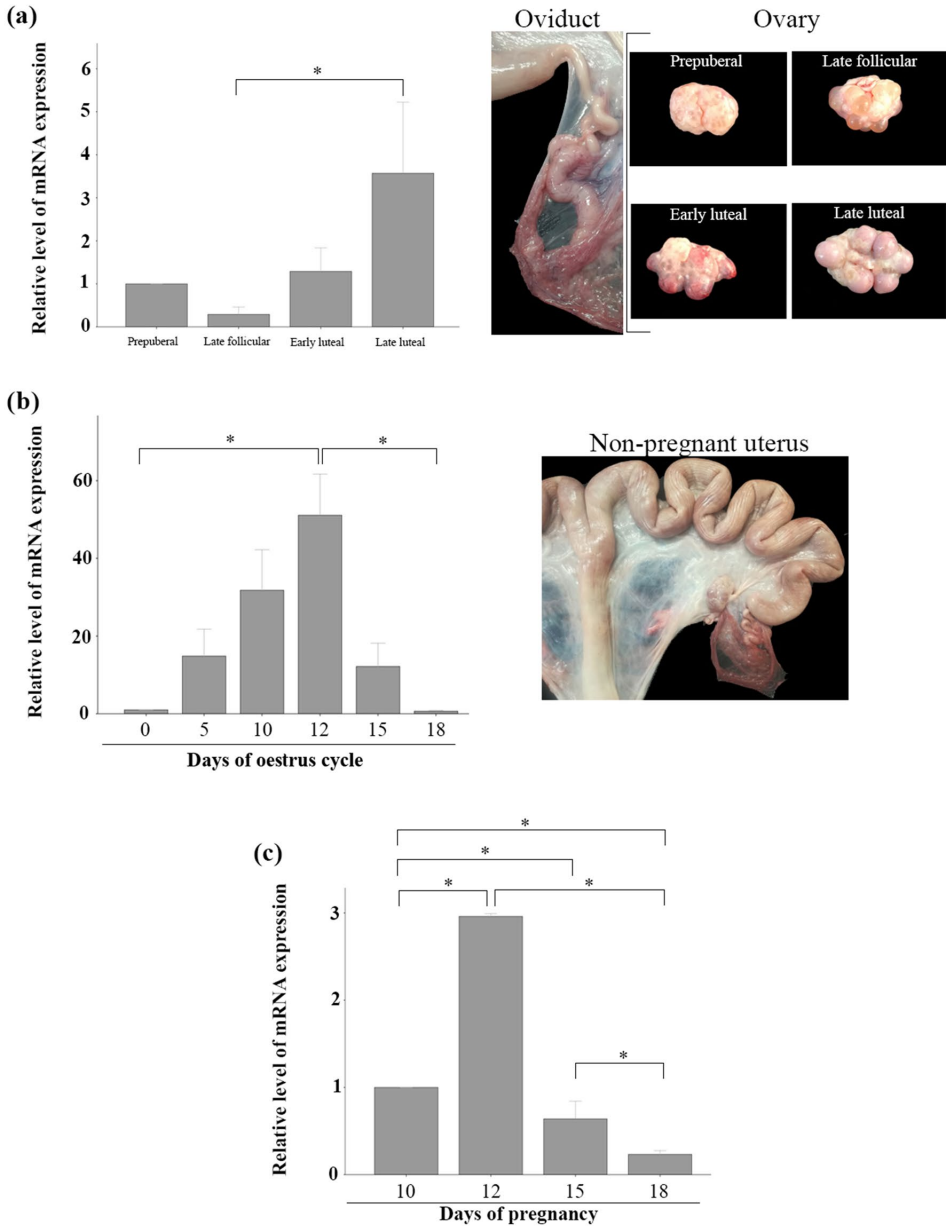
**(a)** Relative level of mRNA expression of haptoglobin in the oviduct in different phases of the porcine oestrus cycle. (*) Between experimental groups indicates significant differences (p = 0.07). **(b)** Relative level of mRNA expression of haptoglobin in endometrium of cyclic sows. Day 0 indicates the ovulation point. (*) Between experimental groups indicates significant differences (p < 0.05). **(c)** Relative level of mRNA expression of haptoglobin in endometrium of pregnant sows. (*) between experimental groups indicates significant differences (p < 0.05). Data are shown as mean ± SEM.

When relative haptoglobin mRNA abundance was quantified by RT-qPCR, the results showed that it is continuously expressed in porcine oviduct throughout the oestrous cycle. However, its expression was significantly lower during the late follicular phase than in the late luteal phase. The expression increased in the early luteal phase, reaching a maximum expression in the late luteal phase (p = 0.07) (Fig. 1a). The expression of haptoglobin mRNA in porcine endometrium was lowest during oestrus (day 0), reaching a maximum level on day 12 of the oestrous cycle (p < 0.05) (Fig. 1b). Moreover, similar results were obtained in pregnant sows (analysed from day 0 to day 18 of pregnancy), in which haptoglobin reached its highest level of expression on day 12 (Fig. 1c).

### Haptoglobin is present in oviductal and uterine fluids

**Figure 2:**
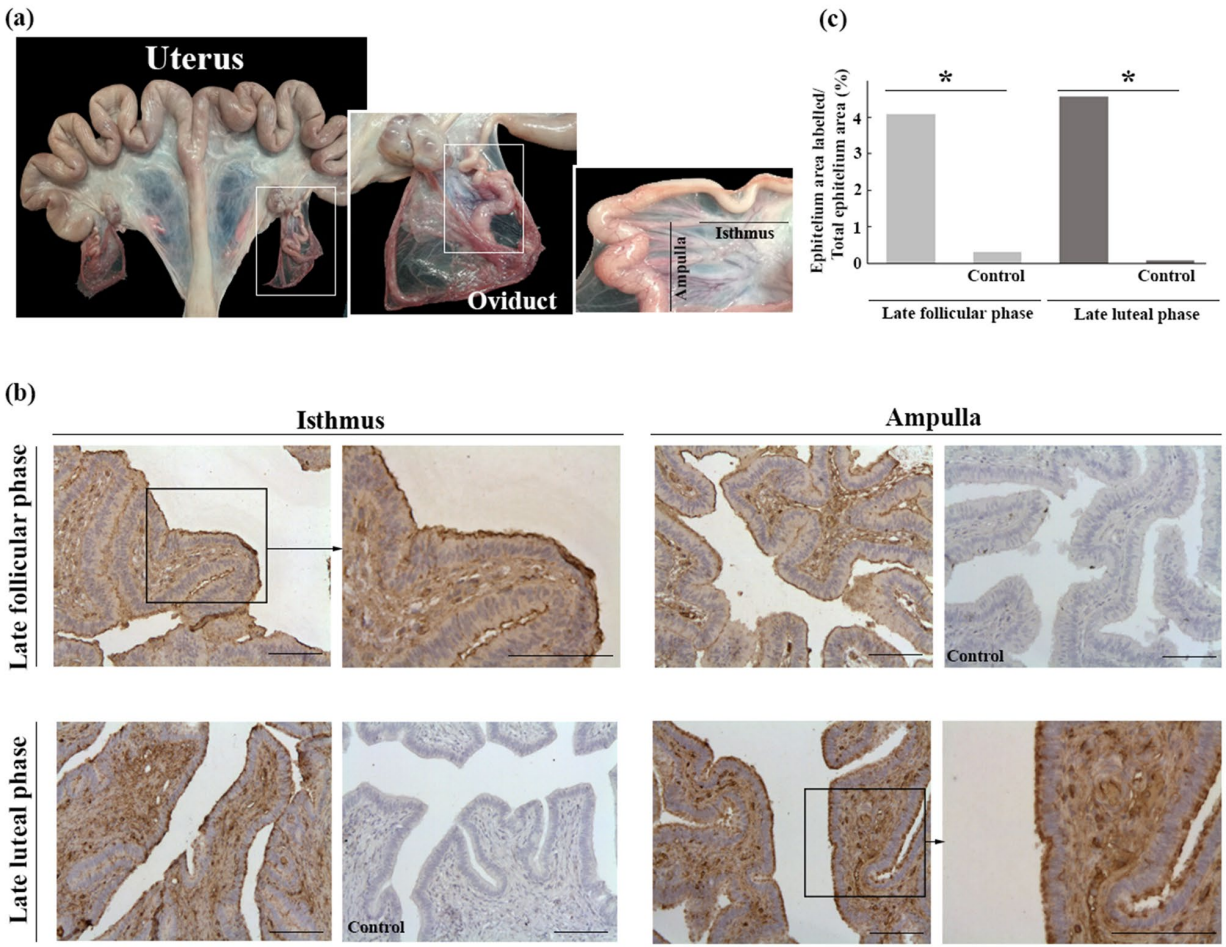
**(a)** Representatives images of a porcine female genital tract including the uterus and oviduct. Samples for the immunohistochemistry analysis were obtained from the ampulla and isthmus of the oviduct. **(b)** Immunohistochemical localization of haptoglobin in porcine oviduct in late follicular and late luteal phases. Haptoglobin labeling was detected at the epithelial cells (intense staining) of the isthmus and ampulla. No staining was observed in the control images (without primary antibody) of the isthmus and ampulla. Scale bar = 100 µm. **(c)** Quantification of the immunohistochemical signal of haptoglobin in late follicular and late luteal phases. Left Y-axis represents the reactive area in the oviductal epithelium with respect to the total epithelial area. (*) Between columns of the same phase indicates significant differences (p < 0.05).

With respect to the origin of haptoglobin, an immunohistochemical analysis of the porcine oviduct (Fig. 2a) detected the protein in the ampulla and isthmus during late follicular and late luteal phases, with a major accumulation in the apical region of the epithelium (Fig. 2b). Both ciliated and non-ciliated cells showed staining in the cytoplasm and on the outer surface of cells. In both phases (late follicular and luteal), a signal was also detected in the lamina propria, mainly in the endothelium of blood vessels and tissue connective cells (Fig. 2b).

The reactive area was quantified in the oviductal epithelium and the total epithelial area. In both the late follicular and late luteal phases, staining was more intense in the oviductal epithelium than in the total epithelial area. This difference was confirmed by statistical analysis, which showed significant differences between each phase and its control (p < 0.05), but no differences were observed when the two phases were compared (Fig. 2c).

**Figure 3:**
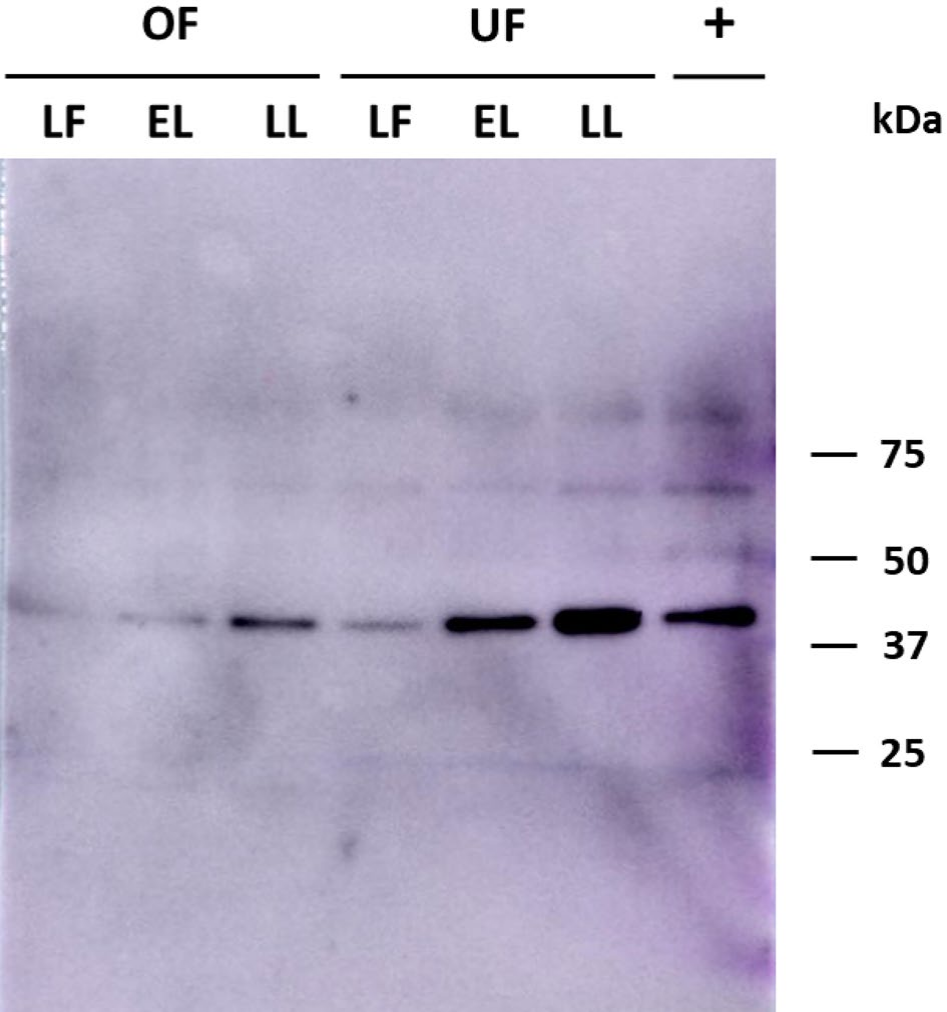
Haptoglobin immunodetection by Western blot in porcine oviductal (OF) and uterine (UF) fluids. A band of ⁓ 45 kDa was detected in all samples. 1) Oviductal fluid from late follicular (LF) 2) Oviductal fluid from early luteal (EL) 3) Oviductal fluid from late luteal (LL) 4) Uterine fluid from late follicular (LF) 5) Uterine fluid from early luteal (EL) and 6) Uterine fluid from (LL) phases 7) Porcine blood serum as a positive control (0.8 µg).

In order to verify the presence of haptoglobin in oviductal and uterine fluids, proteomic analyses were performed in late follicular, early luteal and late luteal fluids. Western-blot analysis showed a single band with a molecular weight ⁓ 45 kDa, according to the positive control (porcine blood serum). The intensity of the bands increased from the late follicular phase to the late luteal phase (Fig. 3).

For the HPLC–MS/MS analysis, samples from different oestrous stages (late follicular, early luteal and late luteal) were analyzed. Bands with a molecular weight of ⁓ 10 kDa, ⁓ 22 kDa, ⁓ 45 kDa, ⁓ 55 kDa and ⁓ 70 kDa were trimmed and analyzed. Haptoglobin peptides were only detected in the ⁓ 45 kDa band (Additional file 1). A total of 20 and 15 different haptoglobin peptides were detected in the oviductal and uterine fluids, respectively (Table 1 and Additional file 2).

**Table 1:**
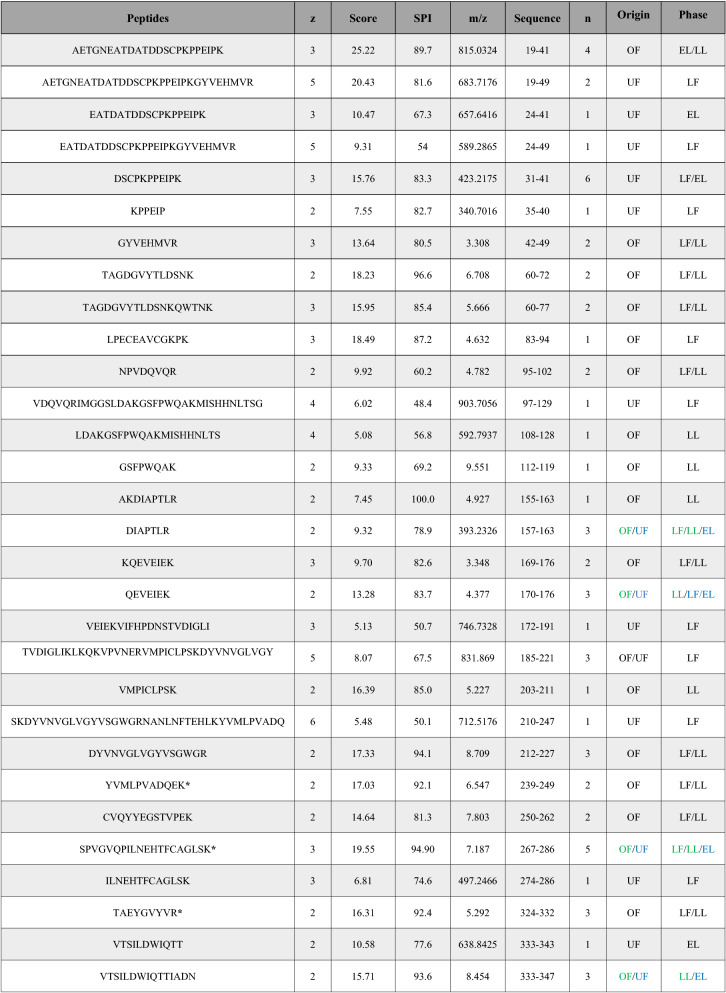
Peptides corresponding to haptoglobin protein detected by HPLC-ESI-MS/MS. When the same peptide was detected several times, the data corresponding to the peptide with the highest score is shown. z: represents the charge of the detected ion or fragment. m/z: mass/charge ratio. Score: score based on signal strength. SPI: scored peak intensity. Sequence: order of appearance of amino acids in the protein. n: number of times that the peptide is detected. Origin: oviductal fluid (OF) or uterine fluid (UF). Phase: stage of the cycle when the peptide was detected: late follicular (LF), early luteal (EL), and late luteal (LL). Detected peptides from the gel stained with PageBlue Protein Staining and trimmed are identified with an asterisk (*). In the origin column black color is used when a peptide was detected in both OF and UF, green color is used for OF and blue color for UF.

### Addition of exogenous haptoglobin improves the developmental competence of porcine embryos

**Figure 4:**
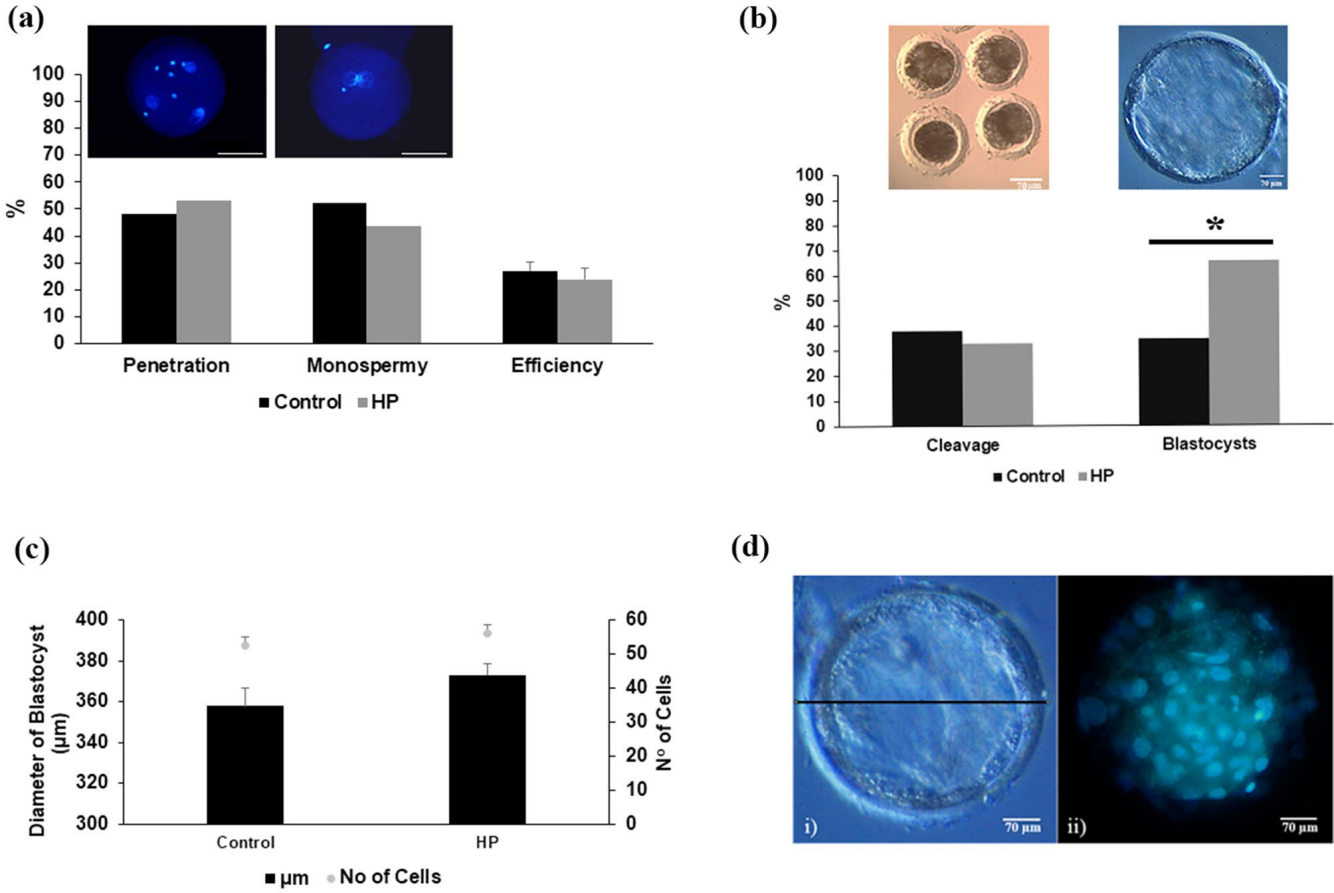
Effect of haptoglobin on in vitro embryo production and development. (**a**) Percentage of penetration, monospermy and efficiency rates during the gametes co-incubation with haptoglobin (n = 7 replicates) (93 and 107 oocytes were evaluated for control and haptoglobin groups, respectively) (**b**) Percentage of zygote cleavage (at 48 h post-insemination) and blastocyst development (at 7 days post-insemination) were evaluated (n = 3 replicates). Blastocyst rate was calculated from percentage of cleaved embryos. (*) between experimental groups indicates significant differences (p < 0.05) (104 and 94 zygotes were evaluated for control and haptoglobin groups, respectively). (**c**) Left Y-axis represents the diameter (µm) in represented and right Y-axis represents the total number of the cells per blastocysts evaluated on day 7 post insemination. The data are expressed as mean ± standard error (37 and 31 blastocysts from control and haptoglobin groups were evaluated) (**d)**, (i) The diameter of the blastocyst was measured using ImageJ® program, and (ii) total number of cells was evaluated from blastocyst stained with Hoechst.

The presence of haptoglobin during co-incubation with gametes had no effect on penetration, monospermy or IVF efficiency rate (p > 0.05) (Fig. 4a). In the case of embryo culture, cell division was similar in both experimental groups (35.60% control vs. 32.97% with haptoglobin; p > 0.05); however, blastocyst development was significantly higher in the haptoglobin group than in the control (37.83% control vs. 64.50% with haptoglobin; p < 0.05) (Fig. 4b).

To determine the quality of the embryos, the diameter of the blastocyst (µm) and the number of cells per blastocyst were evaluated (Fig. 4c-d). In the case of the diameter, the control group values (357.86 ± 8.98 µm) were statistically similar to those of the haptoglobin group (373.13 ± 5.40 µm; p > 0.05) (Fig. 4c). Similarly, the total number of cells per blastocyst was similar in both groups (control: 52.46 ± 2.73 vs. haptoglobin: 56.11 ± 2.50; p > 0.05) (Fig. 4c).

## Discussion

The ideal environment for in vitro embryo development is that which best mimics naturally occurring conditions in the female reproductive tract. Oviductal and uterine fluids are complex and dynamic fluids secreted by the epithelial lining of the reproductive tract and are composed of hormones, enzymes, amino acids, inorganic salts, metabolites, lipids, glycosaminoglycans, proteins, and extracellular vesicles, among others^8,9,11,12,54–57^. However, culture media lacks most of these components. The presence of haptoglobin in the female reproductive fluids of several species^21,40,42,49–53^ suggests that this protein is of some relevance for the modulation of gamete interaction and early embryo development. Our results indicate that this protein is secreted by epithelial cells to the oviduct and uterus lumen and that its expression varies along the oestrous cycle in porcine species. Furthermore, its addition to IVF and in vitro culture (IVC) media improves the efficiency of IVP porcine embryos.

The fact that IVF efficiency is lower than that of in vivo fertilization in most species^25,58^ may be due to failures in gamete maturation, alterations in sperm-oocyte interactions or the deficient development of the recently formed zygote. Most of these processes, which may jointly affect the final outcome of in vitro procedures, take place in the oviduct and uterus under in vivo conditions and, consequently, understanding the factors that affect them is of great importance for further advances to be made in the field of reproductive biology.

In our first approach, haptoglobin expression was analyzed in porcine oviduct and uterus. RT-qPCR showed that haptoglobin was present in the oviduct throughout the oestrous cycle, similar to the results observed in cattle by RT-PCR analysis, which identified it in the peri-oestrous and luteal phases^41^. In rabbit, haptoglobin was found in the oviduct from day 0 to day 3 post-fertilization (coinciding with the embryo’s passage through the oviduct), but was undetectable by day 4^42^. Our results also showed the presence of haptoglobin mRNA in the endometrium of cyclic sows, particularly during the luteal phase; however, Jalali and collaborators^24^ found higher expression levels on day 9 of the cycle, falling by day 12 of the luteal phase. In rabbits, the expression of haptoglobin in the endometrium was already detectable on day 1, increasing up to day 5 and reaching a maximum on day 6 (last day investigated)^42^. In women, haptoglobin expression in the endometrium was seen to be higher during the secretory phase than in the proliferative phase^45^, which agrees with our results, which showed that the haptoglobin level was higher in the late luteal phase than in the late follicular phase. In pig, the late luteal phase encompasses the stage when luteal bodies are formed, and haptoglobin expression seems to coincide with the increased presence of progesterone (secreted by the corpus luteum) and decrease in oestrogen levels. This agrees with the results observed in a study of the porcine transcriptome during the oestrous cycle^59^, and seems to confirm the relationship between haptoglobin and preimplantational embryo development, which occurs in the oviduct and uterus during the luteal phase.

Haptoglobin was also detected in the uterus of pregnant sows, and, in accordance with the results of Jalali, haptoglobin expression was seen to increase during the luteal phase^24^. The increase observed during the late luteal phase might be related with the immune-modulating and angiogenic properties of the protein^45^ and might contribute to endometrial receptivity for conceptus attachment^24^. Our results showed that the expression of haptoglobin increased to reach a maximum on day 12 of pregnancy. However, early embryos in rat^60^ and rabbit^42^ do not express haptoglobin, which is not expressed in the fetal rat liver until day 18 of pregnancy^60^. Our results could be in accordance with the data published for rat^60^, and the decrease in expression in the porcine endometrium after day 12 of pregnancy could be related with the expression of haptoglobin in the embryo from this day. This observation merits further study.

The presence of haptoglobin in the oviductal and uterine fluids was demonstrated by Western-blot and proteomic analyses in different oestrous stages (late follicular, early luteal and late luteal). Western-blot analysis showed the presence of haptoglobin in both fluids with a molecular weight of ⁓ 45 kDa, which is similar to the approximately 40 kDa described in cow oviductal fluid^40^. Studies made in rabbit^43^ and human^45^ uterus confirm this molecular weight. It is evident from our quantitative PCR and Western-blot results that mRNA levels lead to corresponding protein levels throughout the oestrous cycle, with greater expression in the late luteal phase.

The haptoglobin present in oviductal and uterine fluids could be a secretory product of the oviduct and endometrium and/or a transudate of blood serum. To clarify this, an immunohistochemical analysis of the ampulla and isthmus was performed. The results showed an intense immunostaining of the epithelium, confirming that the protein is secreted by the epithelial cells, as previously reported in cattle^40^, although in this species, the immunostaining of the isthmus was less robust compared with that of the ampulla^40^. However, quantification of the signals from the follicular and luteal phases pointed to no differences between the stages.

Pigs are a species with a potential use in biomedicine due to the anatomical, physiological and genetic similarity with human species^61–63^, and, for this and other reasons, ARTs are of great interest for researchers. However, the success of porcine IVF and IVP is far from optimal. Polyspermy and insufficient oocyte cytoplasmic maturation are thought to be the main reasons for the low efficacy of IVF^29^, while the developmental competence of IVP embryos is low compared with that of in vivo embryos^25,26^, which emphasizes the need to improve current in vitro maturation (IVM)-IVF-IVC systems.

Our results point to a higher expression of haptoglobin in both the oviduct and uterus during the luteal phase, which seems to confirm the presence of haptoglobin in the microenvironment that surrounds the pre-implanted embryo and led us to make functional analyses using this protein. The results showed that the supplementation of IVF and IVC media with haptoglobin improves blastocyst development. A significant effect was observed in haptoglobin-incubated blastocyst compared to the control group, and no impairment in blastocyst quality in terms of the total number cells per blastocyst or their size. Several studies have evaluated the supplementation of IVM-IVF-IVC systems with different molecules (reviewed in^30,64^), including natural fluids, such as oviductal fluid^26,65–68^ or exosomes^69^, finding an increase in the efficiency of IVF and/or the quality of embryos. However, in our study, the percentage of improvement achieved in IVP compared to the control group (haptoglobin: 64.50% vs. control: 37.83%) is one of the greater observed differences^70–75^. As indicated, a significant increase was observed in the blastocyst rate when haptoglobin was added, however no effect was observed in the embryo cleavage rate. Thus, these results suggest that haptoglobin helps in embryo development only when it reaches the first divisions but not before, despite its presence. In rabbits, at least, the blastocyst fluid contains haptoglobin although the embryo itself does not express the protein, which indicates that the embryo is able to uptake haptoglobin^42^. Indeed, the uptake of other components by embryos differs from the early embryo to blastocyst stages^76–78^, indicating that embryos can uptake and use components depending on the surrounding conditions or developmental stage. Porcine embryo genome activation occurs at the 4-cell stage^79,80^. Thus, in the light of our results, it seems likely that the intake of haptoglobin from this stage has a beneficial effect on the blastocyst rate.

Haptoglobin can also function as a potent immunosuppressor of lymphocyte function, modulating helper T cells^81^ and probably negatively regulating the inflammatory response^82^. Therefore, its presence in the oviduct and uterus may play an important role in the modulation of maternal-embryonic crosstalk. The presence of haptoglobin in the extraembryonic matrix of rabbit embryos^42^, probably arising from oviductal and uterine secretions since the embryo does not express haptoglobin^42,60^, implies the uptake of maternal haptoglobin into the extra-embryonic matrices as the embryo passes through the oviduct and/or the uterus^42^. The biological significance of the incorporation of haptoglobin into the extra-embryonic matrix remains unknown, although embryo development and embryo implantation might be supported by the intake of maternal haptoglobin^42^.

## Conclusions

In conclusion, our observations demonstrate that haptoglobin is produced and secreted by the epithelium lining the oviduct and uterus during different phases of the oestrous cycle. This data also suggests that it plays an important role in early embryo development, as do other proteins^83–85^, and highlights the importance of developing defined culture media that closely mimics physiological fluids. Future studies will be directed at determining the molecular mechanism of this protein.

## Methods

### Oviduct and uterus collection

Oviducts and uteri from adult and prepubertal females (Landrace x Large White) were collected from a local slaughterhouse (El Pozo Alimentación S.A., Alhama de Murcia, Murcia, Spain) within 15–20 min of death. The phases of the oestrous cycle were defined by ovarian morphology as previously reported^86^. The oviducts were classified as follows: (1) prepubertal: ovary containing follicles 1–2 mm in diameter; (2) late follicular: ovary containing follicles (N = 6 to 12) 8–12 mm in diameter; (3) early luteal: ovary containing from 6 to 12 haemorrhagic corpora; and (4) late luteal: ovary containing from 6 to 12 corpora lutea. Samples were collected for RNA isolation, storing in RNAlater (Sigma-Aldrich®, Madrid, Spain) at -80 ºC until use, immunohistochemistry (samples fixed in 4% paraformaldehyde) and to obtain oviductal fluid and uterine fluid (stored at -80 ºC).

Collection of uterine endometrium was conducted using sexually mature and cyclic crossbred gilts. Gilts (n = 40) were checked for estrous twice daily using intact boars. The onset of behavioural oestrous was considered day 0 and a subset (pregnant; n = 16) were bred on day 0 and again on day 1 using artificial insemination while 24 were not mated (non-pregnant). Non-pregnant gilts were sacrificed on day 0, 5, 10, 12, 15 and 18 while pregnant gilts were sacrificed on day 10, 12, 15 and 18 of early pregnancy. Following humane euthanasia, uteri were removed and endometrial tissue for RNA was excised from the antimesometrial side of the uterine horn and snap frozen in liquid nitrogen and stored at -80 °C as previously described^87^.

### RNA isolation and cDNA synthesis

Total RNA was isolated from samples using TRIzol reagent (Thermo Fisher Scientific, Roskilde, Denmark) according to the manufacturer’s instructions. The quality of total RNA was determined electrophoretically with an RNA 6000 Nano LabChip kit and the Agilent 2100 Bionalyzer (Agilent Technologies). All samples used for the experiments had an RNA integrity number (RIN) > 7^88^. The reverse transcription reaction was performed using MultiScribe Reverse Transcriptase (Thermo Fisher Scientific) using 1 mg total RNA.

### Real time RT-PCR

The relative expression levels of haptoglobin in the prepubertal sow oviduct (used as expression reference), and in late follicular, early luteal and late luteal stages were determined using specific primers for haptoglobin, β-actin (ACTB) and glyceraldehyde-3-phosphate dehydrogenase (GAPDH) used as an internal control (Table 2). The primers were validated using oligonucleotides properties calculator (https://www.sigmaaldrich.com/pc/ui/easy-oligo-home/easyoligo) and BLAST tool (https://blast.ncbi.nlm.nih.gov/Blast.cgi). Three samples were used per group. The oviduct was frozen in liquid nitrogen at slaughterhouse and transport to the lab on ice. Later, Real-time RT-PCR was carried out in tStepOne ThermalCycler (Applied Biosystems, California, USA). The 5X HOT FIREPol EvaGreen qPCR Mix Plus (Solis BioDyne) was used, with SYBR green and normalising against ROX (passive reference dye). Cycle threshold (Ct) values were obtained using OneStep Software (Applied Biosystems). Genomic DNA contamination was avoided by using a DNase treatment step and oligonucleotides designed with their sequence interrupted by intronic regions.

**Table 2:** Primer information for real time RT-PCR amplification of haptoglobin, β-actin (ACTB) and Glyceraldehyde-3-phosphate dehydrogenase (GAPDH) genes.

Gene	bp (f/r)	Sequence	Tm (ºC)	Genbank accession number
Haptoglobin	21 (f)	GAGGCATAAAAGCAGGTGCAG	64	NM_214000
Haptoglobin	22 (r)	GCTGTCATCTGTGGCATCTGTG	64	NM_214000
ACTB	20 (f)	CTGGCGCCCAGCACGATGAA	66	XM_003124280
ACTB	20 (r)	GACGATGGAGGGGCCGGACT	66	XM_003124280
GAPDH	20 (f)	ACCCAGAAGACTGTGGATGG	62	NM_001206359
GAPDH	20 (r)	AAGCAGGGATGATGTTCTGG	60	NM_001206359

### Collection of oviductal and uterine fluid

The oviductal and uterine fluids from 10 to 12 sows from each group (late follicular, early luteal and late luteal stages) were collected and pooled. For oviductal fluid collection, oviducts were separated from the genital tracts and quickly rinsed with 0.4% cetyl trimethyl ammonium bromide (Cetab) (Sigma-Aldrich®, Madrid, Spain) solution and twice in phosphate buffer solution without calcium and magnesium (PBS; Sigma-Aldrich®, Madrid, Spain) and then transferred to Petri dishes on ice to be dissected. After dissection, oviductal fluid was collected by aspiration with an automatic pipette using a tip designed for a maximum 200 μl volume. The samples were centrifuged at 7200 g for 10 min at 4 $^\circ$C to remove cellular debris, and the supernatant was stored at −80 $^\circ$C until use. The uterine fluid was collected by applying pressure from the uterine tubal junction to the end of the horns. The fluid was centrifuged twice for 10 min at 7200 g and 4 $^\circ$C to remove debris before being aliquoted and stored at −80 $^\circ$C until use.

### Western-blot analysis

The protein concentration of oviductal fluid, uterine fluid and blood serum was determined by absorbance spectrophotometry^89^. Samples (0.3 µg of oviductal fluid and uterine fluid from late follicular and late luteal stages) and a positive control (0.8 µg of blood serum) were separated by SDS-PAGE (NovexTM WedgeWellTM 16% Tris–Glycine Gel, Invitrogen, Waltham, Massachusetts, USA) and transferred to PVDF membranes. The transfer conditions were 40 V for 1 h. The membranes were subsequently blocked in 1% TBTS-BSA (bovine serum albumin), overnight, at 4 °C, and probed with rabbit anti-human haptoglobin (DPATB 2047 RH, Creative Diagnostics) at 1:1000 dilution in 1% TBTS-BSA and goat anti-rabbit IgG conjugated with horseradish peroxidase (SC-2400, Santa Cruz Biotechnology, Germany) at a 1:40,000 dilution in 1% TBTS-BSA. After washing, the membranes were incubated with 1 ml Pierce® ECL 2 western blotting substrate (Thermo Fisher Scientific) at room temperature for 5 min. The chemiluminescence signal was acquired by ImageQuant LAS500 (GE Health Life Sciences).

### HPLC–MS/MS analysis

For HPLC–MS/MS analysis 3 to 5 technical replicates were performed, the samples used were: in-gel and in-solution samples from oviductal fluid and in-solution samples from uterine fluid. In the case of in-gel samples, the proteins were stained with Simply BlueTM Safe Stain (Invitrogen, Carlsbad, CA, USA) after SDS-PAGE. In-gel samples were digested following standard procedure. The protein bands were destained, washed and dried using an Eppendorf 5301 vacuum evaporator. They were then incubated with 100 µl of 25 mM ammonium bicarbonate buffer pH 8.5 with 20 mM DTT at 56 $^\circ$C for 20 min, followed by another incubation step with 100 µl of 25 mM ammonium bicarbonate buffer pH 8.5 with 100 mM iodoacetamide for 30 min at room temperature in the dark. After washing one more time, the spots were dried and then incubated with 50 µl of 25 mM ammonium bicarbonate buffer pH 8.5 containing 0.5 µg of Trypsin Gold Proteomics Grade (Promega Corporation, Madison, MI, USA.) and 0.01% ProteaseMax surfactant (Promega Corporation) for 10 min at 4 ºC. Finally, the samples were incubated for at least 3 h at 37 $^\circ$C. The supernatant was collected in a new tube and dried using the vacuum evaporator. In-solution samples were digested by means of the following standard procedure. Samples were dissolved in 100 µl of 50 mM ammonium bicarbonate buffer pH 8.5 with 0.01% ProteaseMax (Promega) (trypsin digestion enhancer), and then incubated with 20 mM DTT at 56 ºC for 20 min and 100 mM IAA for 30 min at room temperature in the dark. Finally, 1 µg of Trypsin Gold Proteomics Grade (Promega Corporation) (approx. 1:100 w/w) was added and left to stand for 3 h at 37 ºC. The reaction was stopped with 0.1% formic acid and the samples were dried using an Eppendorf Vacuum Concentrator model 5301.

The tryptic digests of the samples were separated and analysed with an Agilent 1290 Infinity II Series HPLC (Agilent Technologies, Santa Clara, CA, USA) connected to an Agilent 6550 Q-TOF Mass Spectrometer (Agilent Technologies) using an Agilent Jet Stream Dual electrospray (AJS-Dual ESI) interface.

Dry samples from trypsin digestion were re-suspended in 20 µl of buffer A, consisting of water/acetonitrile/formic acid (94.9:5:0.1). The sample was injected onto an Agilent AdvanceBio Peptide Mapping HPLC column (2.7 µm, 150 × 1.0 mm, Agilent technologies), thermostatted at 55 ºC, at a flow rate of 0.05 ml/min. After injection, the column was washed with buffer A for 5 min and the digested peptides were eluted using a linear gradient 0–40% B (buffer B: water/acetonitrile/formic acid, 10:89.9:0.1) for 90 min followed by a linear gradient 40–95% B for 20 min. The column was equilibrated in the initial conditions for 10 min before every injection.

The mass spectrometer was operated in the positive mode. Profile data were acquired for both MS and MS/MS scans in extended dynamic range mode. The MS and MS/MS mass range was 50–1700 m/z and scan rates were eight spectra/sec for MS and three spectra/sec for MS/MS. Auto MS/MS mode was used with precursor selection by abundance and a maximum of 20 precursors selected per cycle. A ramped collision energy was used with a slope of 3.6 and an offset of -4.8. The same ion was rejected after two consecutive scans.

Data processing and analysis was performed with Spectrum Mill MS Proteomics Workbench (Agilent Technologies). Briefly, raw data were extracted under default conditions as follows: unmodified or carbamidomethylated cysteines; [MH] + 50–10,000 m/z; maximum precursor charge + 5; minimum signal-to-noise MS (S/N) 25; finding 12C signals.

### Immunohistochemistry

Haptoglobin expression was immunohistochemically analyzed in the oviduct. After fixation in 4% paraformaldehyde, tissue samples (n = 3 for late follicular and late luteal phases) were dehydrated, and embedded in paraplast wax (McCormick, Richmond, IL, USA). Sections from total oviduct (5 µm) were placed on Superfrost Plus microscope slides (Menzel, Braunschweig, Germany), which were then deparaffined in xylene and rehydrated through a descending ethanol series followed by distilled water.

Antigen retrieval was performed by boiling the sections in citrate buffer pH 6 antigen retriever (Sigma-Aldrich®, Madrid, Spain) for 30 min. To block endogenous peroxidase activity, the sections were incubated for 20 min in 98% methanol containing 2% hydrogen peroxide solution (30%). After washing with TBS-Tween, the sections were incubated for 90 min in 6% fetal bovine serum (TBS-Tween) at room temperature to eliminate non-specific antibody bindings. Sections were incubated overnight with primary antibody rabbit anti-pig haptoglobin (DPAB2074, Creative Diagnostic, USA) diluted at 1:10 in TBS-Tween at 4 ºC. The sections were washed three times with TBS-Tween and then sequentially incubated with secondary antibody goat anti-rabbit IgG conjugated with horseradish peroxidase (SC-2400, Santa Cruz Biotechnology, Germany) diluted at 1:50 in TBS-Tween. After washing in TBS-Tween, the immunoreactivity was observed by detecting peroxidase activity using 3,3-diaminobenzidine hydrochloride (DAB Kit, Abcam Plc, Cambridge, UK), counterstained with hematoxylin, dehydrated, cleared through xylene, and mounted in dibutyl phthalate xylene (DPX) (BDH Prolabo, VWR International Ltd., Leicestershire, UK). Negative controls were performed following the same procedure without incubation with the primary antibodies. Immunostained sections were examined with a Nikon Eclipse bright-field microscope and captured by a Zeiss Axiophot microscope coupled to a digital camera (Leica DC 500).

The immunohistochemical staining was then quantified with a Leica QWin® work station from a series of 14 digital pictures from three different animals, that were randomly taken from each stained section, considering separately the oviduct portion (ampulla or isthmus), and the functional stage (late follicular and late luteal). For the negative control, seven digital pictures from three different animals were analysed. The threshold for selection of the grey scale levels corresponding to positive staining was defined for the primary antibody by a double-blind selection method (95% coincidence in threshold levels of two independent observers after 3 trials). Staining was expressed as the percentage of immunopositively stained tissue area in each picture.

### In vitro maturation of oocytes

Ovaries from prepuberal animals (n = 25–30 animals per replicate) were collected from a local slaughterhouse (El Pozo Alimentación S.A.), and transported to the laboratory in sterile saline solution at 38.5 $^\circ$C. Once in the laboratory they were washed with 1% Cetab at 38.5 $^\circ$C. The follicles measuring 3–6 mm in diameter were punctured using 10 ml syringes with a sterile 1.2 × 40 mm needle (G18). The porcine follicular fluid (PFF) was collected into 15 ml tubes and placed on a thermal plate (38.5 $^\circ$C), before allowing to incubate for 5–10 min in order to obtain a pellet.

Once formed, the pellet was washed with DPBS (Dulbecco’s Phosphate Buffered Saline Sigma-Aldrich®, Madrid, Spain) at 38.5 ºC and deposited on a Nunclon™ Delta Surface plate (Thermo Fisher Scientific). A stereoscope microscope (Nikon SMZ-10A, Japan) was used to obtain the oocyte-cumulus cell complex (COCs), which were manipulated by means of a prefabricated glass Pasteur pipette, and those with a homogeneous cytoplasm and at least three layers of cumulus cells were selected. Selected COCs (50–55) were incubated in 500 μl of NCSU-37a medium, North Caroline State University-37 (108.73 mM NaCl, 25.07 mM NaHCO_3_, 4.78 mM KCl, 1.19 mM KH_2_PO_4_, 1.19 mM MgSO_4_•7H_2_O, 1.70 mM CaCl_2_•2H_2_O, 5.55 mM glucose, 1 mM glutamine, 12 mM D-Sorbitol, 0.1 IU/I penicillin G, 0.034 mM streptomycin, supplemented with 0.57 mM cysteine, 1 mM dibutyryl cAMP, 5 µg/ml insulin, 50 µM β-mercaptoethanol, 10 IU/ml equine chorionic gonadotropin (eCG; Foligon; Intervet International), 10 IU/ml hCG (Veterin Corion, Divasa Farmavic), and 10% FFP (v/v) after equilibrating for 3 h at 38.5 °C and 5% CO_2_ for 20–22 h in a 4-well plat. Subsequently, the COCs were transferred to 500 μl of the above described NCSU-37b medium (but free of eCG, hCG and dibutyryl AMPc), in which they were incubated for another 20–22 h under the same conditions^90^.

### In vitro fertilization

Seven replicates were carried out for IVF experiments. After the 40–44 h of IVM, the oocytes were mechanically stripped (to remove the *cumulus* cells) and transferred to another well with TALP medium (114.06 mM NaCl, 3.20 mM KCl, 0.50 mM MgCl_2_•6H_2_O, 10 mM sodium lactate, 0.35 mM NaH_2_PO_4_•H_2_O, 5 mM glucose, 25.07 mM NaHCO_3_, 2 mM caffeine, 8 mM calcium lactate•5H_2_O, 1 mg/ml PVA, 0.17 mM kanamycin, 0.003 mM red phenol, supplement with albumin (6 mg/ml BSA) and sodium pyruvate (0.2 mM) after equilibrating for 3 h at 38.5 ºC in 5% CO_2_^90,91^.

Before IVF, the spermatozoa were subjected to a Percoll® selection gradient using 90% Percoll® (Pharmacia, Uppsala, Sweden) and 45% (BTS Acromax® Beltsville Thawing Solution 1:1 percoll 90%) solutions prepared at room temperature. Then, 2 ml of each solution were added (first percoll-90% and then percoll-45%, taking care not to mix the different phases) to a 10 ml conical tube. Finally, 500 μl of pure semen was added to the top of the density gradient and the tube was centrifuged at 700 g for 30 min at room temperature. The resulting pellet was resuspended and centrifuged again at 700 g for 10 min in TALP medium before determining the sperm concentration in a Neubauer cell-counting chamber. Oocytes were co-incubated with 1 × 10^4^ selected spermatozoa per ml (5000 spermatozoa in 500 μl TALP, final concentration). Haptoglobin porcine purified protein (Cat # HGLB12-N-25. Alpha Diagnostic International, USA) (final concentration of 10 µg/ml) was added depending on the experimental group. Oocytes and spermatozoa were incubated for 18 h at 38.5 ºC / 5% CO_2_^90,91^.

Subsequently, IVF output was evaluated by Hoechst stain. Briefly, putative zygotes were washed in PBS supplemented with 1 g/l of polyvinyl alcohol (PBS/PVA) and then fixed in glutaraldehyde (0.5% in PBS) for 30 min. Putative zygotes were washed for 5 min in PBS/PVA and successively stained for 15 min in 1% Hoechst (bisbenzimide H trihydrochloride 33,342, Sigma-Aldrich®)^7^ with PBS in darkness. Finally, zygotes were mounted on slides and evaluated under fluorescence microscopy (Leica® DM4000 Led Germany. 460 / 490 nm). The parameters evaluated were the following: oocyte penetration (swollen sperm heads and/or male pronuclei in the ooplasm; %), monospermy (oocyte with only one swollen sperm head or male pronuclei; %) and IVF efficiency [efficiency, % = final number of putative zygotes (monospermic) per 100 penetrated oocytes in each group].

### In vitro embryo culture

Three replicates were carried out for in vitro embryo culture experiments. Eighteen hours post insemination, the putative zygotes were transferred to supplemented NCSU23a medium (NaCl 108.73 mM, NaHCO_3_ 25.07 mM, KCl 4.78 mM, KH_2_PO_4_ 1.19 mM, MgSO_4_•7H_2_O 1.19 mM, CaCl_2_•2H_2_O 1.70 mM, sodium pyruvate 0.5 mM, sodium lactate 5 mM, L-glutamine 1 mM, taurine 7 mM, penicillin G 0.1 IU/I, streptomycin 0.034 mM, insulin 0.087 mM and β-mercaptoethanol 50 μM, supplemented with hypotaurine 5 mM, BSA-FAF 0.4%, MEM 1% (non-essential amino acids), BME 2% (essential amino acids) and 10 μg/ml of haptoglobin (only for haptoglobin group), and incubated at 38.5 ºC, 5% CO_2_ and 7% O_2_ for 22–24 h. Subsequently, the cleavage percentage was evaluated and only those zygotes that presented 2 to 4 cells were transferred to NCSU23b medium (NaCl 108.73 mM, NaHCO_3_ 25.07 mM, KCl 4.78 mM, KH_2_PO_4_ 1.19 mM, MgSO_4_•7H_2_O 1.19 mM, CaCl_2_•2H_2_O 1.70 mM, D-glucose 5.55 mM, L-glutamine 1 mM, taurine 7 mM, penicillin G 0.1 IU/I, streptomycin 0.034 mM, insulin 0.087 mM and β-mercaptoethanol 50 μM, supplemented with hypotaurine 5 mM, BSA-FAF 0.4%, MEM 1%, BME 2% for another 120 h under the same conditions previously mentioned, to complete a development of 7 days post insemination.

Finally, the blastocysts obtained were photographed (Nikon®D40, Japan, digital camera) under an inverted microscope (Nikon®Diaphot300, Japan). The diameter of the photographed blastocyst was evaluated by ImageJ® program (v.1.48d open software) downloaded from the National Institute of Health (USA). Subsequently, blastocysts were stained by Hoescht (as previously explained for the oocytes) to evaluate the total number of cells per blastocyst using a fluorescence microscope (Leica® DM4000 Led Germany. 460/490 nm).

### Statistical analysis

For real time RT-PCR, the Ct values were analysed using statistical software (Microsoft Office Excel), normalising the values in each sample against those of the housekeeping genes, β-actin (ACTB) and glyceraldehyde-3-phosphate dehydrogenase (GAPDH) used as an internal control. Then, the mean values were compared among the normalised Ct samples, and fold changes were determined. The assumptions of normality and homogeneity of variances were checked by Shapiro–Wilk and Levene tests, respectively. If the tests were fulfilled, a one-way ANOVA followed by a post hoc Tukey test was applied. If the tests were not fulfilled (only for comparison of level of expression of the oviduct under different oestrous stages), a Kruskal–Wallis test was performed. In all cases, the differences were considered statistically significant at p < 0.05.

For immunohistochemistry, the data were first examined using the Shapiro–Wilk test to assess normality distribution, and differences between each phase and its control were compared using an ANOVA test. When ANOVA revealed a significant effect, the values were compared by post hoc test (Tukey). Differences were considered statistically significant at p < 0.05.

For IVF and in vitro embryo production, statistical analyses were performed using IBM® SPSS® Statistics software version 24. All the endpoints (penetration, monospermy, cleavage and blastocyst rates) were represented as percentages, except for the IVF efficiency rate, which was represented as mean ± SEM. Differences between treatments were analysed by Chi-square test. Values were considered significant when p < 0.05.

## Supplementary information

Supplementary Information.

## Data Availability

All data generated or analysed during this study are included in this published article and its supplementary information files.
