# Supplementary material for: Evidence of haptoglobin in the porcine female genital tract during oestrous cycle and its effect on in vitro embryo production
Source: Sci Rep. 2021 Jun 8;11:12041. doi: 10.1038/s41598-021-90810-6 (PMC8187724; doi:10.1038/s41598-021-90810-6)
Supplement: Supplementary file 1 — Supplementary Information. [file 41598_2021_90810_MOESM1_ESM.docx]

**Additional file 1.** SDS-PAGE of porcine oviductal fluid (OF) and porcine blood serum stained with PageBlue Protein Staining. 1) OF from late follicular (LF) (50 µg) 2) OF from late luteal (LL) (50 µg). 3) Swine blood serum (+) (24 µg). Bands marked with a red box (⁓ 10 kDa, ⁓ 22 kDa, ⁓ 45 kDa, ⁓ 55 kDa, ⁓ 70 kDa) were trimmed and analyzed by HPLC-ESI-MS/MS. The band (⁓ 45 kDa) where haptoglobin peptides were detected is shown with an asterisk (*).


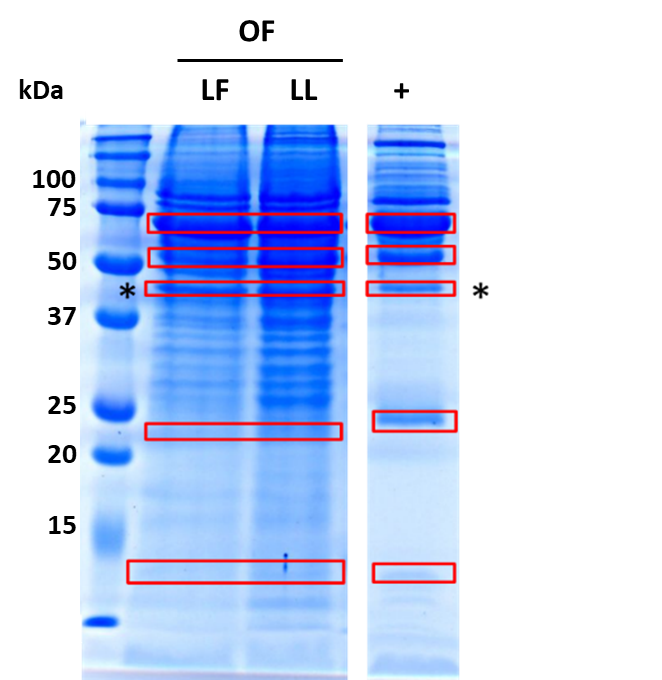


**Additional file 2.** Haptoglobin protein sequence (NP_999165). Peptides detected by HPLC-ESI-MS/MS appear underlined.

1 MRALGAVVAL LLCGQLFAAE TGNEATDATD DSCPKPPEIP KGYVEHMVRY HCQTYYKLRT

61 AGDGVYTLDS NKQWTNKVTG EKLPECEAVC GKPKNPVDQV QRIMGGSLDA KGSFPWQAKM

121 ISHHNLTSGA TLINEQWLLT TAKNLRLGHK NDTKAKDIAP TLRLYVGKKQ EVEIEKVIFH

181 PDNSTVDIGL IKLKQKVPVN ERVMPICLPS KDYVNVGLVG YVSGWGRNAN LNFTEHLKYV

241 MLPVADQEKC VQYYEGSTVP EKKTPKSPVG VQPILNEHTF CAGLSKYQED TCYGDAGSAF

301 AVHDKDDDTW YAAGILSFDK SCRTAEYGVY VRVTSILDWI QTTIADN
